# Pegcetacoplan ‐ a novel C3 inhibitor for paroxysmal nocturnal hemoglobinuria

**DOI:** 10.1002/hsr2.512

**Published:** 2022-04-25

**Authors:** Syeda Tayyaba Rehan, Mahnoor Rehan Hashmi, Muhammad Sohaib Asghar, Muhammad Junaid Tahir, Zohaib Yousaf

**Affiliations:** ^1^ Internal Medicine Dow University of Health Sciences Karachi Pakistan; ^2^ Medicine Lahore General Hospital Lahore Pakistan; ^3^ Internal Medicine Hamad Medical Corporation Doha Qatar

Paroxysmal nocturnal hemoglobinuria (PNH) is a rare, chronic, life‐threatening hematologic disorder due to clonal expansion of mutated bone marrow stem cells,^1^ in which their progeny lose the capacity to bind proteins to the cell surface. Chronic and/or paroxysmal intravascular hemolysis and predilection to thrombosis are caused by the loss of the complement inhibitors CD55 and CD59 on the surface of red blood cells (RBC). A fraction of patients may also develop clinically severe aplastic anemia or myelodysplastic syndrome, as well as hypocellular or dysplastic bone marrow. PNH is characterized by fatigue, dysphagia, abdominal pain, dyspnea, dark urine, and erectile dysfunction.^2^ It predominantly affects Asians between 30 and 59 years. Therapy for PNH is evolving rapidly with the availability of biologic therapies that target the underlying complement‐mediated hemolysis.^3^

The incidence of clinically significant PNH is believed to be between 1 and 10 cases per million individuals, however, this figure might be underestimated because some patients stay undiagnosed. PNH primarily affects adults, males, and females equally. PNH stems from the acquired somatic mutations in hematopoietic stem cells' phosphatidylinositol glycan class A gene (PIG‐A).^4^ Impairment in PIG‐A genes causes the production of erythrocytes that lack glycosylphosphatidylinositols (GPI) anchor proteins such as CD59 (membrane inhibitor of reactive lysis, MIRL) and CD55 (complement decay‐accelerating factor, DAF).^5^ The loss of complement inhibitors (CD55 and CD59) renders red blood cells (RBCs), platelets, and leukocytes vulnerable to hemolysis by membrane attack complex (MAC).^1^ The absence of a nucleus in red blood cells makes them more prone to complement‐mediated lysis among the other marrow progeny.^1^ In PNH, intravascular hemolysis is mediated by complement component‐5 (C5), whereas extravascular hemolysis is complement component‐3 (C3) mediated.^1^ The hemolysis leads to anemia, thrombosis, muscle dystonia, hemoglobinuria, pulmonary hypertension, and increased risk of kidney disease.^1^

Clinical results in 1610 individuals with PNH were published in an international registry.^6^ Nearly everyone was symptomatic (>93%), and many had a poor quality of life, had been hospitalized (23%), or were unable to work (17%). Other frequent manifestations were fatigue (80%), dyspnea (64%), hemoglobinuria (62%), abdominal pain (44%), bone marrow suppression (44%), erectile dysfunction (38%), chest pain (33%), thrombosis (16%), and renal insufficiency (14%). Around half of the patients had neutropenia and/or thrombocytopenia, with a cumulative incidence of pancytopenia in 15% of individuals at 8 years.^7^ PNH is usually diagnosed by flow cytometry that demonstrates a population of granulocytes and RBCs that are deficient in GPI‐linked proteins (eg, CD55, CD59), and bone marrow examination. It is further classified as hemolytic (classic) PNH, subclinical, or PNH with bone marrow failure on the basis of the presence of symptoms (eg, anemia‐related symptoms, thrombosis, pain, organ dysfunction) and findings from bone marrow examination. Surrogate indicators including serum lactate dehydrogenase (LDH) levels and leukocyte clone size differ amongst PNH categories, but they aren't utilized for categorization since they do not correlate well with the severity of disease and bone marrow abnormalities. For patients who present with PNH and severe bone marrow failure (ie, significant leukopenia, thrombocytopenia, and/or dysplasia due to severe myelodysplasia), allogeneic hematopoietic cell transplantation is also considered.^8^

Till May 2021, eculizumab was the frequently prescribed evidence‐based treatment of PNH. Eculizumab is a C5 inhibitor that suppresses intravascular hemolysis by preventing the formation of MAC.^3^ Eculizumab showed benefit against thrombotic complications, pulmonary hypertension, and renal insufficiency and is safe in pregnancy.^4^ However, it fails to block the C3 mediated extravascular hemolysis, leading to anemia requiring blood transfusions.^5^ C5 complement inhibitors (eg, ravulizumab, eculizumab) are well documented in the treatment of hemolytic PNH symptoms such as thrombosis, pain, and organ failure. The major purpose of complement inhibitor therapy is to relieve PNH‐related symptoms (such as fatigue and dyspnea), eliminate transfusion dependency, avoid thromboses, and relieve pain. Clinical responses are frequently followed by a drop in serum lactate dehydrogenase (LDH) levels to less than 1.5 times the upper limit of normal. Despite the fact that these drugs essentially remove the danger of thrombosis, more than half of patients still experience mild to severe symptoms, and up to 20% require further treatment. A retrospective analysis of 141 patients treated with eculizumab showed that 72% of individuals remained anemic, and 36% required more than one transfusion annually.^5^ Importantly, complement inhibitors do not mitigate symptoms and complications of PNH‐associated bone marrow failure, such as aplastic anemia (AA) or myelodysplastic syndrome (MDS), which will ultimately require stem cell transplantation.^8^ Ravulizumab is occasionally favored to eculizumab because of its convenience of use, cheaper overall cost, and fewer occurrences of pharmacokinetic breakthrough hemolysis, although its effectiveness and toxicity are generally equivalent. Ravulizumab has a 4‐fold longer terminal half‐life than eculizumab, allowing for longer treatment intervals (eg, 8 weeks vs 2 weeks, respectively) and lower yearly costs. Both drugs bind to the same epitope on C5 and prevent C5 convertase from cleaving C5 into C5a and C5b, inhibiting complement action.^8^

A new drug, Pegcetacoplan, showed promising results and was approved by the food and drug administration (FDA), on 14th May, 2021 to treat PNH. Pegcetacoplan is a novel C3 inhibitor administered as a subcutaneous infusion twice weekly and can inhibit both intravascular and extravascular hemolysis.^9^ It is a targeted C3 inhibitor consisting of two 15‐amino acid cyclic peptides conjugated to a linear polyethylene glycol molecule. Pegcetacoplan binds to C3 and inhibits its activation.^3^ It cleaves the C3 fragments and curbs the C3 mediated extravascular hemolysis in PNH patients.^5^ The cleavage of C3 is supervened by blockage of the entire complement system as it serves as a point of convergence for all complement pathways.^5^ In an International 16 week‐long multicenter study, Pegcetacoplan showed improvements in hemoglobin levels by 2.97 g per deciliter in PNH patients.^10^ Half‐life of pegcetacoplan is 8 days. The drug significantly increases the serum C3 level and reduces the bilirubin level within 16 weeks.^9^ It decreased the reticulocyte count but did not affect lactate dehydrogenase levels.^3^ In patients on eculizumab, a temporary suspension of pegcetacoplan resulted in a drastic decrease in hemoglobin and serum C3 levels.^3^ In most studies, pegcetacoplan was administered along with eculizumab as a combined therapy to avoid the risk of hemolysis with a sudden switch of treatment.^9^ Pegcetacoplan was superior to eculizumab for reducing transfusion dependence and lessening fatigue in a study, with PNH patients who had Hb <10.5 g/dL despite prior eculizumab therapy.^10^ Patients were randomly assigned to pegcetacoplan monotherapy (41 patients) or eculizumab monotherapy (42 patients) after a four‐week run‐in phase in which all patients received pegcetacoplan with eculizumab (39 patients). Pegcetacoplan patients experienced a nearly 4 g/dL rise in hemoglobin levels, higher transfusion independence (85 vs 15%, respectively), and an improved tiredness score (FACIT‐Fatigue score) at week 16 compared to eculizumab patients.^10^ The most common adverse events during treatment with pegcetacoplan and eculizumab groups were injection site reactions (37 vs 3%, respectively), diarrhea (22 vs 3%), breakthrough hemolysis (10 vs 23%), headache (7 vs 23%), and fatigue (5 vs 15%).^10^

The common adverse effects of pegcetacoplan are headache, diarrhea, fatigue, and respiratory tract infections.^9^, ^10^ Castro et al reported eight severe treatment‐emergent adverse events in two of the PNH patients that were administered with pegcetacoplan during the trial. These included three urinary tract infections and a single event of pyrexia, pancreatitis, and lower gastrointestinal hemorrhage.^3^ Increased levels of alanine aminotransferase (ALT) and aspartate aminotransferase (AST) were also observed.^3^ In less than 1% of participants, allergic reactions were reported.^9^ Patients should be vaccinated against encapsulated bacteria at least 2 weeks before initiation of pegcetacoplan to avoid infections caused by encapsulated organisms.^9^ Breastfeeding is contraindicated and can be resumed 40 days after the last dose.^9^

The Median survival rate of PNH patients increased from 10 to 20 years in the early 2000s after the approval of eculizumab.^1^ Previously, Hillmen et al^11^ reported a median survival of approximately 10 years in their report of 80 patients with thrombosis as a predominant cause. Age >50 years, significant cytopenias upon diagnosis, severe infection, thrombosis, and renal failure were included among bad prognostic factors with the Asian population more likely to suffer aplastic anemia, and American patients are more likely to have thrombosis. As per a clinical study by Kelly et al, there was a significant gap between the 5‐year survival rate amongst the patients treated with complement inhibitors and the patients who did not receive any complement suppressing therapies. The 5‐year survival rate of PNH patients is observed around 66.8% which is significantly lower than the 5‐year survival rate of 95.5% for the patients treated with complement inhibitor (ie, eculizumab).^12^ This recent drug, pegcetacoplan, if used appropriately, can hopefully elevate the life standard in PNH patients by reducing the life‐threatening complications of the disease. However, the survival data of this drug is very limited at this point of time, but with other approaches to blocking complement activation including monoclonal antibodies to other complement proteins, peptide inhibitors, small molecule inhibitors, and decoy receptors are also in various stages of preclinical development, promising data on pegcetacoplan makes this drug vital for the treatment of PNH. Since PNH is a chronic disease with significant morbidity and mortality, this novel drug can potentially replace high‐risk therapies like blood transfusions. Side effects like urinary tract infections, injection site‐local ailments, pyrexia, and gastrointestinal hemorrhage are a concern. Authors believe that selecting suitable patients before initiating pegcetacoplan will make a significant difference in the overall management of PNH, since treating intravascular hemolysis, as well as the prevention of extravascular hemolysis with pegcetacoplan, may result in better control of the disease. We recommend that ALT and AST levels be routinely monitored during drug administration. Other effects of this drug during pregnancy, pediatric age group, and on patients with underlying comorbidities should be a part of phase IV surveillance. For pregnant women with PNH who require anticomplement therapy, decreased maternal mortality and morbidity and improved fetal outcomes should also be studied with this novel drug. More extensive studies are needed to explore this drug's efficacy and safety, given the limited data available.

## FUNDING

The authors have no financial or proprietary interest in the subject matter of this article.

## CONFLICT OF INTEREST

The authors have no conflict of interest.

## AUTHOR'S CONTRIBUTIONS

Conceptualization: Syeda Tayyaba Rehan, Mahnoor Rehan Hashmi.

Data curation: Syeda Tayyaba Rehan, Mahnoor Rehan Hashmi, Zohaib Yousaf.

Investigation: Syeda Tayyaba Rehan, Mahnoor Rehan Hashmi, Muhammad Junaid Tahir.

Methodology: Syeda Tayyaba Rehan, Mahnoor Rehan Hashmi, Muhammad Junaid Tahir.

Resources: Muhammad Sohaib Asghar.

Software: Muhammad Sohaib Asghar.

Validation: Syeda Tayyaba Rehan, Muhammad Sohaib Asghar, Muhammad Junaid Tahir.

Visualization: Muhammad Sohaib Asghar, Muhammad Junaid Tahir.

Project administration: Muhammad Junaid Tahir, Zohaib Yousaf.

Supervision: Zohaib Yousaf.

Funding Acquisition: Zohaib Yousaf.

Writing ‐ original draft: Syeda Tayyaba Rehan, Mahnoor Rehan Hashmi.

Writing ‐ review and editing: Muhammad Sohaib Asghar, Muhammad Junaid Tahir, Zohaib Yousaf.

All authors have read and approved the final version of the manuscript. The corresponding author has full access to all of the data reviewed in this study and takes complete responsibility for the integrity of the data reported.

## ETHICS STATEMENT

Not required as this is a viewpoint.

## Data Availability

No data associated with this submission.
